# Prostaglandins in Cancer Cell Adhesion, Migration, and Invasion

**DOI:** 10.1155/2012/723419

**Published:** 2012-02-29

**Authors:** David G. Menter, Raymond N. DuBois

**Affiliations:** ^1^Department of Cancer Biology, The University of Texas MD Anderson Cancer Center, Houston, TX 77030, USA; ^2^Department of GI Medical Oncology, The University of Texas MD Anderson Cancer Center, Houston, TX 77030, USA

## Abstract

Prostaglandins exert a profound influence over the adhesive, migratory, and invasive behavior of cells during the development and progression of cancer. Cyclooxygenase-2 (COX-2) and microsomal prostaglandin E_2_ synthase-1 (mPGES-1) are upregulated in inflammation and cancer. This results in the production of prostaglandin E_2_ (PGE_2_), which binds to and activates G-protein-coupled prostaglandin E_1–4_ receptors (EP_1–4_). Selectively targeting the COX-2/mPGES-1/PGE_2_/EP_1–4_ axis of the prostaglandin pathway can reduce the adhesion, migration, invasion, and angiogenesis. Once stimulated by prostaglandins, cadherin adhesive connections between epithelial or endothelial cells are lost. This enables cells to invade through the underlying basement membrane and extracellular matrix (ECM). Interactions with the ECM are mediated by cell surface integrins by “outside-in signaling” through Src and focal adhesion kinase (FAK) and/or “inside-out signaling” through talins and kindlins. Combining the use of COX-2/mPGES-1/PGE_2_/EP_1–4_ axis-targeted molecules with those targeting cell surface adhesion receptors or their downstream signaling molecules may enhance cancer therapy.

## 1. The Prostaglandin Pathway

**Figure 1:**
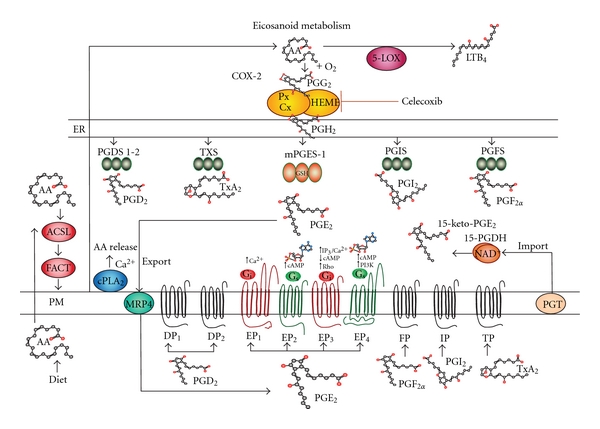
Eicosanoid metabolism. Arachidonic acid (AA) is an essential dietary fatty acid that is transported into cells and stored in membrane phospholipids. First AA is coupled to acyl-CoA by acyl-coenzyme A synthetases (ACLS). Fatty acyltransferases (FACT) then insert AA into membrane phospholipids. Cytoplasmic phospholipase A2 (cPLA2) releases AA from membrane phospholipids after agonist stimulation. In turn, free AA is converted to prostaglandin G_2_ (PGG_2_) and then prostaglandin H_2_ (PGH_2_) by cyclooxygenases (COXs). PGH_2_ then becomes a substrate for a variety of PG synthases. These PG synthases are identified by the specific prostaglandin each one produces, namely, PGD_2_ synthases (PGDSs), PGE_2_ synthases (PGESs), (PGF_2*α*_) synthase (PGFS), PGI_2_ synthase (PGIS), or TxA_2_ synthase (TXS). Both COX-2 and microsomal PGE synthase-1 (mPGES-1) are elevated in tumors. Export involves multidrug resistance-associated protein 4 (MRP4). In the extracellular milieu, PGs bind to G-protein-coupled receptors identified as DP1, DP2, EP1-4, FP, IP, and TP. Among these, EP receptors interact with G-stimulatory (Gs) or G-inhibitory (Gi) proteins stimulating downstream signals such as cAMP, Ca^2+^, inositol phosphates or IP3/Ca^2+^, and Rho. Catabolism involves uptake by PG transporter (PGT) and inactivation by NAD+ dependent 15-hydroxyprostaglandin dehydrogenase (15-PGDH).

Prostaglandins (PGs) and other eicosanoids are bioactive lipids that impact normal development, tissue homeostasis, inflammation, and cancer progression [1]. Prostaglandins are derived from the 20-carbon chain fatty acid, arachidonic acid (AA) stored in the plasma membrane of cells [2, 3]. As a storage mechanism, dietary AA is coupled to CoA molecules by acyl-coenzyme A (acyl-CoA) synthetases [4]. In turn, fatty acyltransferases utilize arachidonyl-CoA donor molecules to insert AA into membrane phospholipids [2, 3]. Membrane phospholipids generally retain AA until an appropriate stimulus catalyzes its release by phospholipase A2 [5–8] (Figure 1). 

Once released, free AA serves a substrate for cyclooxygenases (COX) 1 or 2 (~72 kDa; Figure 1). Cyclooxygenases are mixed function oxidase enzymes that first peroxidate AA to form a hydroperoxy endoperoxide that links two oxygen molecules across carbons 9 and 11, prostaglandin G_2_ (PGG_2_). As the second coordinate enzymatic function, COXs reduce a hydroperoxy-group at carbon 15 of PGG_2_ to form prostaglandin H_2_ (PGH_2_) [9, 10]. As a rate-limiting product in this pathway, PGH_2_ serves as the substrate for a variety of PG synthases. These PG synthases include various isoforms of prostaglandin D_2_ (PGD_2_) synthases (PGDS) [11], prostaglandin E_2_ (PGE_2_) synthases (PGES) [12–16], and prostaglandin F_2*α*_ (PGF_2*α*_) synthase (PGFS) [17]. PGH_2_ can also be synthesized into prostacyclin (PGI_2_) by its own separate synthase [18, 19] (PGIS) or thromboxane A_2_ (TxA_2_) by its synthase (TXS) [20]. In the case of inflammatory and carcinogenic activity, increased expression of COX-2 and microsomal PGE synthase-1 (mPGES-1) both occur to amplify the accumulation of PGE_2_ in tumors [21–26].

Once synthesized, prostanoids are transported into the extracellular microenvironment by specific multidrug resistance associated proteins (MRPs). These MRP molecules contain 12-transmembrane spanning domains in the plasma membrane and two cytosolic ATP-binding/hydrolysis sites [27]. Among these export molecules, MRP4 is a 160 kDa protein that acts as the primary transporter for PGs.

Once exported to the microenvironment, prostanoids bind to G-protein coupled receptors that contain 7 transmembrane spanning domains. These PG receptors include DP1, DP2, EP1-4, FP, IP, and TP that are classified according to their ligand specificity [28]. There are four EP receptors that require G-stimulatory (Gs) or G-inhibitory (Gi) proteins to initiate downstream signals such as cAMP, Ca^2+^, and inositol phosphates [29]. More specifically, EP1 regulates Ca^2+^ flux; EP2 and EP4 both increase cAMP levels; whereas EP3 decreases cAMP, increases IP3/Ca^2+^, and activates Rho. These signaling pathways frequently initiate transcription or crosstalk with other signal transduction pathways [30–32]. Prostaglandins can also interact with nuclear receptors. Peroxisome proliferator-activated receptors (PPARs) are nuclear receptors that also bind PGs and complex with retinoic X receptors (RXRs) to initiate gene transcription [33, 34].

The catabolism of PG occurs as a two-step uptake and then inactivation process. PGs are taken up by a 12 transmembrane domain glycoprotein known as a PG transporter (PGT) [35–37]. After PGE_2_ is transported across the plasma membrane, it is enzymatically catabolized by NAD+ dependent 15-hydroxyprostaglandin dehydrogenase (15-PGDH) causing inactivation [36, 38, 39]. Two NAD+-15-PGDH protein monomers (29 kDa) form enzymatically active complexes by dimerization. Interactions with biologically active prostaglandins containing hydroxyl groups at carbon 15 are inactivated by conversion to 15-keto catabolites. The levels of both PGT and 15-PGDH are decreased in cancer leading to the accumulation of PGE_2_ in tumor tissues [35, 36, 39, 40]. The accumulation of PGE_2_ in the developing tumor microenvironment promotes tissue reorganization, angiogenesis, as well as cell adhesion, migration and invasion through the basement membrane barrier.

## 2. Prostaglandins and Cadherins: Making and Breaking Cell-Cell Contacts

**Figure 2:**
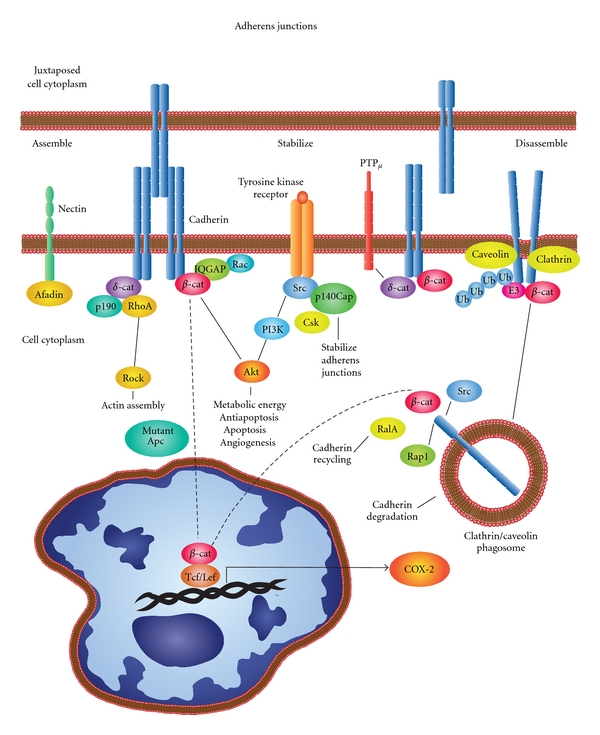
Dynamic adherens junctions. Prostaglandins influence the assembly, stabilization, and disassembly of cell-cell junctions. E-cadherins form Ca^2+^-dependent transmembrane adhesion complexes between adjacent cells (Figure 2). Cytoplasmic regulatory proteins include *α*-catenin, *β*-catenin, IQGAPs scaffold proteins that interact with Rho GTPases to alter morphology and migration. Alternate interactions involve *δ*-catenin, p190, and RhoA influencing actin assembly. Together, Src and p140Cap influence C-terminal Src kinase (Csk) activity stabilizing cell-cell interactions as well as similar activity by receptor protein tyrosine phosphatase mu (PTP*μ*). Nectins-afadin complexes also cooperate with cadherins and integrins to regulate cell-cell adhesion. Disassembly of cadherin complexes involves either caveolin- or clathrin-mediated endocytosis and phagosome formation. Inside-out vesicles contain cadherin on the inside and *β*-catenin and Src exposed to the cytoplasm. When these vesicles interact with Ras-related protein A (RalA), cadherins are recycled. Whereas, interactions with Ras-proximate-1/Ras-related protein-1-(Rap1-)GTPase and E3 ubiquitin ligase followed by ubiquitinization result in proteosomal degradation that prepares cells for migration. The loss of E-cadherin in conjunction with elevations in COX-2 occurs during the transformation and adenoma formation in the presence of Apc mutations causing aberrant *β*-catenin signaling. Subsequent interactions with T-cell factor/lymphoid-enhancer-factor-(TCF/LEF-) can cause increases in COX-2 expression.

Prostaglandins play an important role in wound healing and tissue reorganization [41–46]. The ordered structure of epithelial and endothelial tissues involves the cadherin family of molecules [47–51]. In many epithelial and vascular tissues, prostaglandins influence the formation and loss of cell-cell contacts [52–56]. In vascular tissues for example, prostaglandins potentiate vascular endothelial VE-cadherin-dependent cell adhesion [57]. In the case of epithelial tissues, epithelial E-cadherins are structurally organized into adherens junctions that form extracellular Ca^2+^-dependent transmembrane adhesion complexes between adjacent cells (Figure 2).

In the cytoplasm of epithelial cells, binding proteins mediate interactions between the E-cadherin cytoplasmic domain and the actin cytoskeleton that can trigger a variety of signaling processes [51, 58–60]. Dynamic analyses have revealed that *α*-catenin shuttles between cytoplasmic multiprotein complexes of *β*-catenin/E-cadherin or actin filaments [61]. *β*-catenin/E-cadherin interactions are regulated by IQGAPs that are actin-binding scaffold proteins [56–58]. IQGAPs interact with Rho GTPases and transmit extracellular signals that influence morphological and migratory cell behavior [62–64]. Alternate interactions through *δ*-catenin involve p190 and RhoA [65]. Additional adherens junctions stabilization pathways also exist. One of these pathways includes the involvement of Src and p140Cap. p140Cap regulates Src activation by C-terminal Src kinase (Csk) activity in epithelial-rich tissues that is phosphorylated after cell matrix adhesion [66–68]. Similarly, receptor protein tyrosine phosphatase mu (PTP*μ*) has a cell-adhesion molecule-like extracellular segment and a catalytically active intracellular segment involved in regulating cell-cell interactions [69, 70]. Nectins-afadin complexes also regulate cell-cell adhesion cooperatively with cadherins and integrins [71, 72]. Dynamic maintenance of cell-cell junctions in epithelial and endothelial tissues is critical to their functions as permeability or protective barriers and their continuous turnover as stress interfaces with the surrounding micro- or macro environment.

In order for epithelial cells to migrate, they must break their adhesive contacts with neighboring cells [56, 73]. The disassembly of cadherin containing adherens junctions involves internalization through endocytosis that result in the formation of phagosomes [51]. Internalization occurs by either caveolin-mediated endocytosis or clathrin-mediated coated pits [74–76]. Once cadherin-containing phagosomes are internalized, the extracellular domain resides inside the vesicles that form. At the same time, *β*-catenin and Src that are bound to the cytoplasmic domain of E-cadherin at the plasma membrane end up on the outside of these vesicles. Interactions of these vesicles with Ras-related protein A (RalA) drive cadherin recycling [77]. Interactions between E-cadherin with Ras-proximate-1/Ras-related protein 1 (Rap1)-GTPase, E3 ubiquitin ligase followed by ubiquitinization lead to proteosomal degradation [78–80]. Thus, the internalization and turnover of E-cadherin enables cells preparing to migrate with the ability to break their adhesive contacts between adjacent cells.

Breaking adhesive contacts occurs during tissue homeostasis, angiogenesis, and cancer progression in vascular or epithelial tissues and is a very rapid process based on live cell imaging [49, 81, 82]. In the case of epithelial tissues, their normal uniform structure typically becomes disorganized or dysplastic and then anaplastic during cancer progression. Disorganization in these tissues typically requires breaking cell-cell junctions maintained by cadherins such as E-cadherin [83]. In some cases this is mediated by prostaglandins. In squamous cell carcinoma, for example, chronically UV-irradiated SKH-1 mice sequentially lose E-cadherin-mediated cell-cell contacts as lesions progress from dysplasia to SCCs [53] (see Table 1). In these studies, the loss of E-cadherin levels was inversely associated with increased PGE_2_ synthesis. Furthermore, the loss of E-cadherin involved the EP2 receptor and was reversed by indomethacin or potentiated by the EP2 receptor agonist butaprost [53].

Other epithelial tumors exhibit a similar loss of E-cadherin as COX-2/PGE_2_ levels increase [84, 85]. This loss of E-cadherin is often accompanied by an elevation of vimentin that is a characteristic of cells becoming more migratory during epithelial-to-mesenchymal transition (EMT) [86]. This EMT involving COX-2 is observed in human colon cancers [87]. The loss of E-cadherin in conjunction with elevations in COX-2 occurs during the transformation of rat intestinal epithelial (RIE) cells [88] and during adenoma formation in Apc^*Min*⁡∖+^ that exhibit aberrant *β*-catenin signaling [89] or during gastrulation involving the Snail pathway in Zebra fish [90]. The COX-2 promoter contains a novel functional T-cell factor/lymphoid enhancer factor (TCF/LEF) response element that responds directly to Wnt/*β*-catenin signaling [91]. Regulation involving these pathways in some cases may be reversed. For example, caveolin-1-mediated suppression of COX-2 can occur via a *β*-catenin-Tcf/Lef-dependent transcriptional mechanism [92]. Overall, it is becoming clear that tissue homeostasis, reorganization, angiogenesis, and malignant transformation rely on very rapid dynamic making or breaking of cell-cell junctions centered on cadherin family of molecules. In most cases, epithelial tissues are strengthened by the synthesis and deposition of a basement membrane.

## 3. The Basement Membrane Barrier

**Figure 3:**
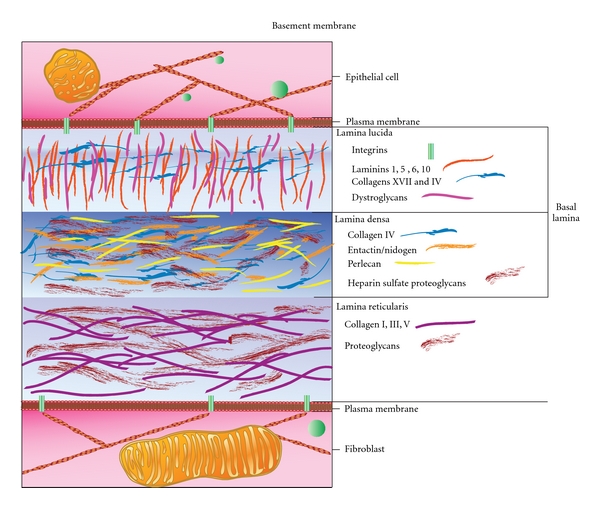
Basement membrane. The basement membrane underlies the typical cellular epithelium or vascular endothelium and consists of two thin structural layers. One layer is the basal lamina made by epithelial or endothelial cells. The second layer is the reticular lamina made by fibroblasts. Electron microscope data show that the basal lamina consists of a clear lamina lucida next to epithelial cells and an opaque lamina densa. The lamina lucida contains integrins, laminins (1, 5, 6 and 10), and collagen XVII, as well as type IV collagen, and dystroglycans. The lamina densa contains type IV collagen fibers, entactin/nidogen-1, perlecan, and heparan sulfate proteoglycans. The reticular lamina contains collagens I, III, and V and various proteoglycans. Invasion through the basement membrane requires the expression of many different cell surface adhesion receptors and matrix degrading enzymes.

Malignancies frequently develop from epithelial precancerous lesions that are initially confined to organ ducts or the epithelial strata of tissues. The pathologic conversion to cancerous lesions often involves malignant cells breaching or invading through the fibrous sheet-like barrier of the basement membrane (Figure 3) [93]. Prostaglandins are involved in the synthesis, homeostasis, turnover, and structural reorganization of the basement membrane [94, 95]. The basement membrane underlies the typical cellular epithelium or vascular endothelium and consists of two thin structural layers. The first layer consists of a basal lamina that is synthesized by epithelial or endothelial cells that differ in their respective characteristics [96]. The second layer is the reticular lamina made by fibroblasts, among other surrounding cells [97]. At the electron microscope level, the basal lamina is subdivided into a clear lamina lucida directly under the epithelial cells and a structurally opaque lamina densa [98, 99]. The lamina lucida contains protein and carbohydrate complexes at the cellular interface consisting of integrins, laminins (5, 6 and 10), and collagen XVII, as well as type IV collagen, laminin 1, and dystroglycans [97, 100, 101]. The lamina densa is a meshwork of type IV collagen fibers, entactin/nidogen-1, as well as perlecan, along with hydrous polysaccharide-rich gels of heparan sulfate proteoglycans. The reticular lamina contains collagens I, III, and V that form a heterogeneous network of fibers and a variety of proteoglycans [97]. Some basement membrane structures also contain pores that allow for the passage of cells [97]. The basement membrane is extensively remodeled during inflammatory responses [102, 103] or becomes disorganized in tumor vasculature [104] and in various cancers [105]. Thus there are a large variety of molecules encountered during invasion through the basement membrane that require the expression of many different cell surface adhesion receptors including integrins, cell surface proteoglycans, and tetraspanins.

## 4. Integrins

**Figure 4:**
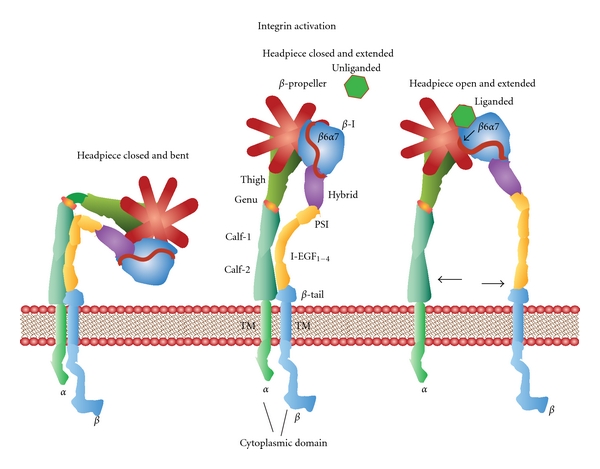
Integrins. Integrins are transmembrane glycoprotein adhesion receptor complexes consisting of *α* and *β* subunits. The *α* subunit contains a seven-bladed *β*-propeller head domain connected to a leg support structure made of a thigh, a calf-1, a calf-2, a transmembrane, and a cytoplasmic domain that mediates ligand specificity. The *β* subunit contains an N-terminal plexin-semaphorin-integrin (PSI) domain, a hybrid domain, a *β*-I domain, four cysteine-rich epidermal growth factor (EGF) repeats, a transmembrane, and a cytoplasmic domain that interacts with the cell cytoskeleton. The N-terminal *β*-I domain of a *β* subunit inserts into the *β*-propeller domain of an *α* subunit forming a headpiece complex. The formation of integrin receptor complexes depends on divalent cation (i.e., Ca^2+^, Mn^2+^, Mg^2+^) that bind to metal-ion-dependent adhesion site (MIDAS) motifs in the *α* subunits and adjacent to MIDAS (ADMIDAS) motifs in *β* subunits. Three conformation states exist for *α* and *β* subunit complexes. (1) The unliganded conformation has a closed headpiece and a bent receptor structure with the EGF domains of the *β*-subunit touching the calf-1-calf-2 domains of the *α*-subunit. (2) The headpiece remains closed, but structural changes in the *β*-subunit EGF domains cause a separation from the calf-1-calf-2 domains of the *α*-subunits causing an extended structure. (3) Conformational changes in the *β*_6_-*α*_7_ loops expose the ligand-binding site along with a complete separation of the *β*-subunit from the calf-1-calf-2 domains in the *α*-subunit. These conformational changes engage the specific integrin headpiece with its ligand.

Mammalian integrins generate heterodimeric transmembrane glycoprotein adhesion receptor complexes consisting of *α* and *β* subunits (Figure 4) [106–109]. Alpha-numeric designations are applied to 18 known *α* subunits (*α* 1–11,D,E,L,M,V,W,X) and 8 *β* subunits (*β* 1–8) available to form pairs in this class of molecules. Each selective pairing recognizes a different ICAM, ligand, or protein substrate in the basement membrane or extracellular matrix [110, 111]. The *α* subunit dictates the ligand specificity by virtue of a seven-bladed *β*-propeller head domain connected to a leg support structure made of a thigh, a calf-1, a calf-2, a transmembrane, and a cytoplasmic domain [107, 112]. The *β* subunit interacts with the cell cytoskeleton and contains an N-terminal plexin-semaphorin-integrin (PSI) domain, a hybrid domain, a *β*I domain, four cysteine-rich epidermal growth factor (EGF) repeats, a transmembrane, and a cytoplasmic domain [107, 112]. In many cases, the N-terminal *β*-I domain of a *β* subunit inserts into the *β*-propeller domain of an *α* subunit (*α*1, *α*2, *α*10, *α*11, *α*L, *α*M, *α*X, and *α*D) to form a bulbous-binding headpiece complex [112]. The formation of integrin receptor complexes depends on divalent cation (i.e., Ca^2+^, Mn^2+^, Mg^2+^) that bind to metal-ion-dependent adhesion site (MIDAS) motifs in the *α* subunits and adjacent to MIDAS (ADMIDAS) motifs in *β* subunits found in the N-terminus of these receptors [107, 111–113]. Together the joined *α* and *β* subunit termini form an N-terminal headpiece [111].

Three conformation states exist for *α* and *β* subunit complexes [114–116]. The first conformation is usually unliganded with a closed headpiece and a bent receptor structure. In this case, the EGF domains of the *β*-subunit are juxtapositioned directly against the calf-1-calf-2 domains in the support leg of the *α*-subunit while the headpiece bends inward toward the plasma membrane [107, 111–113, 116]. Second, the integrin complex headpiece remains closed, but structural changes in the *β*-subunit EGF domains cause them to separate from the calf-1-calf-2 domains of the *α*-subunits and extend away from the plasma membrane [111, 114–116]. Third, altered conformation in the *β*6-*α*7 loops exposes the ligand-binding site while the *β*-subunit completely separates from the calf-1-calf-2 domains in the support leg of the *α*-subunit. These cooperative conformational changes in the heterodimer structures enable the full engagement of a specific integrin headpiece with its ligand [111, 114–116]. These conformational changes can occur during the regulation of “outside-in signaling” [117, 118] or alternatively “inside-out signaling” [112, 119].

## 5. Outside-In Signaling

**Figure 5:**
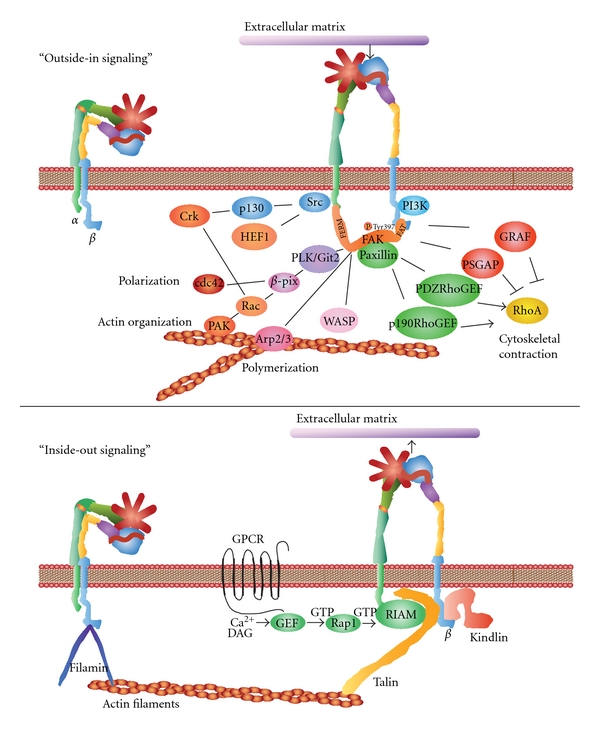
“Outside-in” and “Inside-out” signaling. The “outside-in” binding of ECM ligands to cell surface integrins stimulates conformational changes that activate focal adhesion kinase (FAK). FAK then is autophosphorylated on Tyrosine 397 near the catalytic domain, which binds Src. FAK contains a central kinase domain bordered by FERM (protein 4.1, ezrin, radixin, and moesin homology) domain at the N-terminus and a focal adhesion targeting (FAT) sequence at the C-terminus. Activated Src interacts with human enhancer of filamentation1 (HEF1) and p130 CRK-associated substrate (p130CAS) scaffold proteins that help to positively regulate Src-FAK-Crk interactions with Rac. FAK also activates (PKL/Git2)-*β*-Pix complexes and *β*-pix then serves either as an exchange factor for Cdc42 or a scaffold protein to promote signaling via Rac and p21-activated protein kinases (PAK). FAK also interacts with actin-related proteins (ARP2 and ARP3) which is regulated by the Wiskott-Aldrich Syndrome Protein (WASP). ARP2/ARP3 initiates the polymerization of new actin filaments. FAK also influences actin contraction and polarization through another GTPase protein, Rho. The regulation of Rho GTPase hydrolysis of GTP (active) to GDP (inactive) form occurs through the opposing activities of guanine nucleotide exchange factor (GEFs). GTPase regulator associated with FAK (GRAF) and p190RhoGAP blocks actin cytoskeleton changes. In contrast, PDZRhoGEF and p190RhoGEF both serve to activate Rho. “Outside-in signaling” transfers integrin-mediated external signals to the inside of cells.“Inside-out signaling” depends on talin and kindlin. Both talin and kindlin contain FERM (4.1/ezrin/radixin/moesin) domains and a highly conserved C-terminal F3 domains. Talins bind *β* integrin, actin through the C-terminus, and also vinculin. Kindlins bind integrins, the cell membrane, and various actin adaptor proteins like migfilin, or integrin-linked kinase (ILK). Talin activation occurs through G-protein-coupled receptors that increases cytoplasmic Ca^2+^ and diacylglycerol. This activates GEF function in conjunction with Ras-proximate-1/Ras-related-protein-1-(Rap1-) GTPase. Rap1 then binds to Rap1-GTP-interacting adaptor molecule (RIAM). RIAM recruits talin to the membrane and the *α* and *β* integrin cytoplasmic domains. Kindlin interacts with *β* integrin cytoplasmic domain stabilizing the activated state of the integrin complex. “Inside-out signaling” strengthens adhesive contacts and the appropriate force necessary for integrin-mediated cell migration, invasion, ECM remodeling, and matrix assembly.

Similar to conventional cell surface signal transducing receptors, integrins bind ligands and transmit information in an “outside-in signaling” (Figure 5) [111, 112]. “Outside-in signaling” behavior typically involves the engagement of integrins with the extracellular matrix or ICAM surface receptors [111, 118–121]. When external factors bind to exposed ligand binding site on integrins this results in conformational changes described in the previous section. Most ECM proteins exhibit multivalent or recurrent molecular patterns, which trigger integrin clustering. As cells engage the repetitive patterns in the ECM, these events occur simultaneously thereby activating intracellular signals. The myriad of different extracellular signals that cells encounter in their microenvironment mediates cell polarity, cytoskeletal structure, adhesion, migration, invasion, gene expression, cell survival, and proliferation.

In the case of “outside-in signaling” initiated by ECM proteins, a single ligand-binding event can trigger integrin activation, but repetitive regularly spaced molecular patterns provide a more effective stimulus [122, 123]. This type of mechanoreception has been explored using nanopatterned molecular printing techniques that form regular cRGDfK patch spacings on a polyethylene glycol background matrix [122–125]. These adhesion-dependent sensory mechanisms lead to signal transduction inside the cell by the activating multiple pathways. Focal adhesions are often formed as a result of cell interactions with the ECM substrata, which initiate signal transduction via kinase cascades and other mechanisms.

## 6. Integrins and Focal Adhesions

Focal adhesions were first recognized in Rous sarcoma virus-transformed normal rat kidney cells using an antitumor serum specific for pp60src, as a speckled pattern of fluorescence on the ventral surface (Figure 5) [126]. Focal adhesion kinase (FAK) is a well-studied integrin-activated protein tyrosine kinase (PTK) [127, 128]. FAK was identified as a pp125 tyrosine-phosphoprotein in untransformed chicken embryo cells that increased in pp60v-src-transformed chicken embryo cells [129]. FAK is nonmembrane associated cytoplasmic protein that is autophosphorylated on Tyrosine 397 located at the juncture of the N-terminal and catalytic domains, which directs SH2-dependent binding of pp60src [130]. FAK contains a central kinase domain flanked by FERM (protein 4.1, ezrin, radixin, and moesin homology) domain at the N-terminus and a focal adhesion targeting (FAT) sequence at the C-terminus, which drives localization to focal adhesions [131, 132]. Upon interacting with the cytoplasmic domains of integrins, autophosphorylated FAK interacts with numerous proteins recruited to focal adhesions. It can affect actin organization through the phosphorylation of paxillin [133, 134]. Paxillin phosphorylation by FAK on tyrosine residues 31 and 118 creates binding sites for the Src homology-2 (SH2) domains of adaptor proteins Crk, Csk, and Src [133]. Human enhancer of filamentation1 (HEF1) and p130 CRK-associated substrate (p130CAS) are scaffold proteins that help to positively regulate Src-FAK-Crk interactions [135, 136]. Paxillin can also interact with paxillin kinase linker- (PKL/Git2-) *β*-pix complexes [137]. *β*-pix functions as an exchange factor for Cdc42 or serves as a scaffold protein to promote signaling via the Rho family GTPase Rac and p21-activated protein kinases-(PAK) [137]. FAK-mediated regulation of Cdc42 and Rac activity asserts control over the extension of lamellipodia and cell migration as well as cellular polarization.

As another pathway influenced by FAK, interactions with actin-related proteins (ARP2 and ARP3) either occur directly or are regulated by the Wiskott-Aldrich Syndrome Protein (WASP) [132]. ARP2/ARP3 closely resembles the structure of monomeric actin. ARP2/ARP3 complexes serve as nucleation sites for new actin filaments [138]. When RP2/ARP3 complexes bind to the sides of preexisting actin filaments, they initiate the polymerization of new filaments at a uniform 70° degree-angle during cytoskeletal rearrangements [138]. These molecular activities help organize and expand growing cytoskeletal meshworks of actin filaments.

FAK also influences actin contraction and polarization through another GTPase protein, Rho. The regulation of Rho GTPase hydrolysis of GTP (active) to GDP (inactive) form occurs through the opposing functions of GTPase-activating proteins (GAPs) or guanine nucleotide exchange factors (GEFs) [139]. GAPs make GTP a better substrate for nucleophilic attack thereby lowering the transition state energy for hydrolysis to GDP, inactivating Rho. In contrast, GEFs stimulate the release of GDP from Rho and accelerate the binding of GTP, thereby activating Rho. Among the Rho-inactivating GAP proteins is one that binds to the C-terminal domain of FAK, GTPase regulator associated with FAK (GRAF) to block actin cytoskeleton changes [140]. Another GAP protein, p190RhoGAP, can bind to complexes with p190RasGAP and FAK that alter the cytoskeleton [141]. In contrast, PDZRhoGEF and p190RhoGEF both serve to activate Rho. This activation promotes focal-adhesion turnover and their relocalization within the cell along with cell migration [142, 143].

## 7. Prostaglandins and Focal Adhesion Kinase

 “Outside-in” stimulation by adhesion to ECM also stimulates PG pathway activity and FAK activity (Figure 5). When Hela or NIH3T3 cells are allowed to adhere to ECM, elevations in COX and PKA stimulate the formation of actin bundles that contain myosin II and associate with small focal adhesions and increase cell motility [144]. Similarly, stimulation of Raw264.7 cells with bovine type I collagen increased cyclic-AMP response element-binding protein (CREB) binding to DNA along with COX-2 expression that was reversed by inhibition of FAK [145]. Fluid shear stress stimulation of mechanoreceptors and RDGS mediated disruption of fibronectin adhesions-induced formation of focal adhesions and promoted the upregulation of COX-2 and PGE_2_ release [146]. Similarly, mechanostimulation of osteoblasts activated FAK and PGE_2_ release via integrin stimulation, which increased F-actin fiber formation, causing increased cell stiffness [147, 148]. Furthermore, HEF-1 adaptor proteins that positively regulate interactions with FAK are upregulated by PGE_2_ and stimulate cancer cell migration [149].

Prostaglandins have a profound impact on FAK, immune cells, and cancer. This can occur by stimulation with a variety of PGs. For example, in 293-EBNA (Epstein-Barr nuclear antigen) cells stably expressing prostaglandin F_2*α*_ (PGF_2*α*_) receptors 1 or 2, stimulation with PGF_2*α*_ causes morphological and cytoskeletal changes [150]. The phosphorylation of FAK occurs in association with Rho-mediated morphological and cytoskeletal changes within two minutes, highlighting the rapidness of this process [150]. This FAK-mediated response to PGF_2*α*_ has also been observed in HEK293 cells [151] and endometrial adenocarcinoma cells [152]. Prostaglandin E_2_ is also a strong stimulus for FAK activity. In hepatocellular carcinoma cells for example, PGE_2_ increases the phosphorylation and synthesis of FAK in a dose-dependent manner [153]. Thus PG ligand binding to cognate GPCRs can also initiate “inside-out signaling”.

## 8. Inside-Out Signaling

“Inside-out signaling” depends on a intracellular activators (Figure 5) [119]. These intracellular activators include proteins such as talin or kindlins [120, 154]. There are two talin isoforms and three kindlin isoforms identified thus far [154]. Both talin and kindlin contain FERM (4.1/ezrin/radixin/moesin) domains and a highly conserved C-terminal F3 domain [154]. Talins contain binding sites for several *β* integrin cytodomains, a highly conserved C-terminal actin-binding site and also VBS (vinculin-binding site) [119, 120]. Kindlins contain *β* integrin cytodomain-binding sites in their F3 domains, membrane-binding domains and a C-terminus that interacts with integrins, various actin adaptor proteins like migfilin, or integrin-linked kinase (ILK) [120, 154]. The activation process is thought to begin following stimulation of G-protein-coupled receptors that cause increases in cytoplasmic Ca^2+^ and diacylglycerol, followed by GEF activation in conjunction with Ras-proximate-1/Ras-related protein 1 (Rap1)-GTPase [119, 120]. Rap1 then binds to Rap1-GTP-interacting adaptor molecule (RIAM) [155]. RIAM is the believed to recruit talin to the membrane and the *α* and *β* integrin cytoplasmic domains [119]. Alternatively, talin interacts with PIPKI*γ*/PIP2 and then is cleaved by calpain [119]. Kindlin also interacts with the *β* integrin cytoplasmic domain stabilizing the activated state of the integrin complex [119]. “Inside-out signaling” strengthens integrin-mediated adhesion with extracellular ligands that transfers the appropriate force necessary for cell migration, invasion, ECM remodeling, and matrix assembly [119].

## 9. Prostaglandins, Integrins, and Angiogenesis

**Figure 6:**
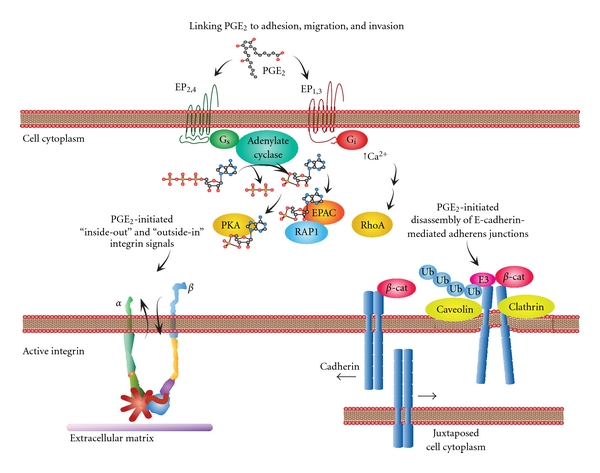
Linking PGE_2_ to adhesion, migration, and invasion. Prostaglandin E_2_ elicits profound changes in tumor cells that result in the disassociation of cadherin-mediated cell connections. This is accompanied by the establishment/turnover of integrin-mediated interactions with extracellular matrix during adhesion and subsequent migration and invasion. Stimulation of EP2 or 4 receptors leads to the activation of adenylate cyclase and results in the production of cyclic adenosine monophosphate (cAMP) from adenosine triphosphate (ATP). The accumulation of cAMP in the cell cytoplasm activates protein kinase A (PKA) and the phosphorylation of downstream targets. This accumulation of cAMP can also activate exchange protein activated by cAMP (Epac). The activation of Epac may involve the interactions with Rap1 and subsequent downstream signals that influence adhesion, migration, and invasion. The activation of EP1 and EP3 leads to Ca^2+^ influx and the activation of Rho-mediated signal transduction that influences cadherin function during the disassociation of cadherin-based adhesive contacts or integrin interactions with the extracellular matrix contacts.

Prostaglandins are known to regulate cellular interactions with extracellular matrix and angiogenesis as early events in cancer progression [1] (Figure 6). The overexpression of COX-2 in rat intestinal epithelial cells was shown to increase adhesion to ECM and inhibit apoptosis which was reversed by sulindac sulfide (a COX inhibitor) [88]. COX-2 also plays a key role in endothelial cell migration and tube formation that relies on interactions with ECM during angiogenesis, which was reversed by NS398 (a COX-2 inhibitor) [156]. Prostaglandin E_2_ plays an important role in stimulating the angiogenic behavior of endothelial cells [157–162]. By contrast, PGE_1_ (alprostadil) inhibits angiogenesis *in vitro* and *in vivo* in the murine Matrigel plug assay [163]. Much of the migratory and invasive behavior of endothelial cells is regulated by signal transducing integrins that initiate changes in cellular shape, adhesion, and motility. For example, endothelial cell migration involves *α*_V_ *β*_3_ (vitronectin) and *α*_5_ *β*_1_ (fibronectin) integrin function, COX-2, the genesis of cAMP involving protein kinase A [164, 165]. This promotion of integrin *α*_V_ *β*_3_ integrin-mediated endothelial cell adhesion, spreading, migration, and angiogenesis appears to occur through COX-2-prostaglandin-cAMP-PKA-dependent activation of the small GTPase Rac [165–167]. Others also confirmed the involvement of *α*_3_ *β*_1_ receptors [168]. Distinct integrins such as *α*_6_ *β*_1_ (laminin) or *α*_1_ *β*_1_ and *α*_2_ *β*_1_ (collagen) receptors are also involved in the migration and invasion of endothelial cells during angiogenesis [169–175]. These studies highlight the role of PG-initiated responses by endothelial cells that involve integrins during the angiogenesis.

## 10. Prostaglandins, Integrins, and Tumor Cell Invasion

Tumor cells also migrate and invade through the basement membrane in response to stimulation by PGE_2_ (Figure 6). For example, PGE_2_ treatment of LS-174T human colorectal carcinoma cells leads to increased motility and changes in cell shape that involves stimulation of the prostaglandin EP4 receptor [176]. In another colon cancer cell line, CaCo2 cell adhesion to type I collagen via *α*_2_ *β*_1_ integrins was stimulated by PGE_2_ and inhibited by COX-2 inhibitors [177]. Similarly, colon cancer cells expressing *β*_1_ integrin levels along with COX-2 inhibition decreased adhesion and migration on ECM [178]. In another study using breast cancer cells, laminin receptor (*α*_3_ *β*_1_) binding to laminin-peptide PA-22 was reduced by PGE_2_ receptor antagonist (LEO101) [179]. Similarly, the suppression of integrin *α*_ 3_ *β*_1_ in breast cancer cells reduced COX-2 gene expression and inhibited tumorigenesis and invasion [168]. In the case of lung cancer, FN stimulated cell proliferation through an *α*_5_ *β*_1_ (fibronectin) integrin-mediated process in conjunction with increases in COX-2 and PGE_2_ biosynthesis that was blocked by NS-398 (a COX-2 inhibitor) [180]. The upregulation of COX-2 also induces tumor cell invasion in models of pancreatic cancer [181]. Other eicosanoids also influence integrin-mediated adhesion and invasion [182, 183]. Collectively, these studies highlight the importance of PGs during integrin-mediated adhesion, migration, and invasion through extracellular matrices by tumor cells.

## 11. Prostaglandins and CNN Proteins

Prostaglandins also regulate the production of matricellular proteins of the CCN family (CYR61/CTGF/NOV) that are emerging as major contributors to chronic inflammatory diseases and regulators of ECM [184]. CCN is an acronym that describes the first three protein family members identified out of six total: **C**YR61/CCN1 (cystein-rich 61;[185]), **C**TGF/CCN2 (connective tissue growth factor; [186]), and **N**OV/CCN3 (nephroblastoma overexpressed; [187]). The other family members consist of structurally conserved secreted multitasking Wnt-inducible secreted proteins (WISP-1/CCN4, WISP-2/CCN5, and WISP-3/CCN6) [188]. Each family member interacts with a specific subset of integrins and can be induced by PGE_2_ depending on the cellular context [184]. In many instances, cell stimulation involving CCNs can alter the production of matrix metalloproteinases [184]. CCN proteins regulate cell adhesion, migration, proliferation, and inflammatory responses that are influenced by PGs [184].

## 12. Prostaglandins and Cell Surface Proteoglycans

Proteoglycans are very heavily glycosylated proteins on the surfaces of cells that heavily influence cell signal transduction and behavior [94, 189–194]. Proteoglycans exert profound control over various aspects of wound healing, angiogenesis, and cancer spreading [192, 195]. The fundamental proteoglycan unit contains a “core protein” and one or more covalently coupled glycosaminoglycans [190, 191]. Coupling occurs through a serine residue to a saccharide bridge found in the glycosaminoglycan. Glycosaminoglycan carbohydrate structures include chondroitin sulfate, dermatan sulfate, heparin sulfate, and keratin sulfate. Proteoglycans fall into two major categories based on size. Small proteoglycans range in molecular weight between 36 to 66 kDa and include decorin, biglycan, testican, fibromodulin, lumican, syndecan, and glypican. Large proteoglycans achieve molecular weights between 136 to 470 kDa and include versican, perlecan, neurocan, and aggrecan within this category. Proteoglycans cooperate dynamically with integrins and growth factors to local adhesion sites or signal complexes to integrate of both external and internal signals [196]. Together with prostaglandins, proteoglycans facilitate adhesion and migration and tubulogenesis by primary endothelial cells and promote phosphorylation of signaling molecules such as Akt and Src [94, 197]. Prostaglandins in concert with proteoglycans also promote the recruitment of stromal cells from the bone marrow to the developing tumor microenvironment [198, 199]. These include CXCL12, CXCR4, and S100A4 producing fibroblasts that involve signaling through a COX-2/PGE_2_-EP3/EP4-dependent pathway [199]. Similarly, the combined effect of prostaglandins and proteoglycans regulates the transition from immature dendritic cells (iDCs) to mature DCs (mDCs) [198]. In breast cancer cells, prostaglandins and proteoglycans stimulate invasion across a basement membrane and induces synthesis of specific heparin-binding splice variants of vascular endothelial cell growth factor (VEGF) [200]. In like fashion, the malignant transformation of intestinal epithelial cells induces the production of VEGF that involves Ras pathway activation [160]. Among the proteoglycans, perlecan in particular plays an integral role in extracellular matrix deposition in response to PGE_2_ [201]. Perlecan is also upregulated during tumor-associated angiogenesis [202], which can be inhibited by decreasing perlecan synthesis [203]. As a whole, proteoglycans work together with prostaglandins to regulate tumor growth and angiogenesis.

## 13. Prostaglandins and Tetraspanins

Tetraspanins form a family of 33 membrane proteins that contain 4 transmembrane-spanning domains [204]. They play important roles in cell adhesion, motility, invasion, immunity, and tumor progression [205–209]. Among these tetraspanins, CD151, CD9, Tspan12, and KITENIN are most known for their role in cancer [205–208, 210]. Tetraspanin CD151 interacts with laminin-binding integrins *α*6*β*1 and *α*6*β*4 to regulate signal transduction activity during growth, migration, invasion, and metastasis [211, 212]. Tetraspanin CD9 in cooperation with cell-surface Ig superfamily proteins, EWI-2 and EWI-F acts to suppress tumorigenesis [213–215]. Tspan12 interacts with a disintegrin and metalloprotease 10 (ADAM10) to initiate protumorigenic functions [216, 217]. Also, KAI1 COOH-terminal interacting tetraspanin (KITENIN) contributes to tumor invasion and metastasis in human colorectal cancers [210] and gastric cancer [218]. In the case of interactions with prostaglandins, prostaglandin F_2_ receptor-associated protein (FPRP) is frequently involved in binding to cancer cell tetraspanins [219, 220]. However, the direct regulation of this class of adhesion related molecules by prostaglandins or eicosanoids remains unknown.

## 14. Recent Advances in Prostaglandin and Adhesion-Based Cancer Therapy

Since (COX-2) is the rate-limiting enzyme in prostaglandin synthesis, it is an effective intervention point for inhibitors [221]. It is well documented that elevated COX-2 levels drive chronic inflammation and carcinogenesis [1]. Clinical and epidemiologic studies clearly demonstrate a significant benefit from inhibiting COX-2 in colon cancer [221, 222]. Unfortunately, COX-2 inhibition is associated with cardiovascular toxicity in a subpopulation of patients at high risk for cardiovascular disease [221, 222]. Nonetheless, this pathway remains an excellent target, based on very strong evidence that the upregulation of COX-2-mediated inflammatory mediators mediates many different cancers [1].

Selective COX-2 inhibition can also initiate a shunt of AA-based substrates to the 5-lipoxygnease (5-LOX) pathway [223], Based on these and other findings, a number of dual pathway inhibitors have been developed that appear to exhibit less toxicity [224–227]. Licofelone is a 5-LOX/COX inhibitor that was developed to treat inflammation and osteoarthritis [228, 229]. In osteoarthritis clinical trials, licofelone inhibits COX and 5-LOX and has low GI toxicity [230, 231]. In another osteoarthritis study, licofelone reduced osteoarthritis symptoms and less cartilage loss by MRI than naproxen [232]. Although developed and tested in osteoarthritis patients, cancer prevention is also an important target. In a lung carcinogenesis mouse model, for example, licofelone showed a dose-dependent inhibition of Cox-2 and 5-Lox and proliferating cell nuclear antigen (PCNA) staining in concert with an increase in apoptosis [233]. An overall reduction in GI toxicity in combination with enhanced anti-inflammatory activity makes these new inhibitors a promising class of compounds for the prevention and treatment of cancer.

Another approach is to specifically target specific points in the proinflammatory and procarcinogenic mPGES1-PGE_2_-EP1-4 axis of the COX-2 pathway [221]. Inhibition of the inducible mPGES-1 has received significant attention [14, 15, 22, 23, 234]. In mouse models, EP(1) and EP(3) receptor antagonists ONO-8713 and ONO-AE3–240, but not the EP(4) antagonists ONO-AE3-208 and AH 23848, inhibited medulloblastoma tumor cell proliferation [235]. In Apc^*Min*⁡∖+^ models of colon carcinogenesis, by contrast, the genetic deletion of mPGES-1 significantly protected against azoxymethane-induced colon cancer [236]. In these studies genetic the deletion mPGES-1 reduced tumor multiplicity by ~80% and tumor load by 90% [236]. Also in a syngeneic mouse model of bone cancer, mPGES-1 enhances tumor growth and associated pain [237]. These studies emphasize the importance of mPGES-1 as a target for cancer prevention and therapy.

As a target further downstream, methods to decrease the accumulation of PGE_2_ in tumors are also a potential target option. In this case, treatment may include enhancing the metabolic turnover of PGs by 15-PGDH. This may require upregulation by reversing histone deacetylase-mediated silencing of 15-PGDH [39]. These approaches are not as well developed as others but remain viable options for reducing prostaglandin-associated inflammation and cancer treatment.

As a target even further downstream, the development of EP selective receptor antagonists has seen extensive focus [238, 239]. In mouse models, ONO-AE3-208, an EP4 receptor antagonist significantly reduced metastasis [240]. Another EP4 antagonist is being tested as an inhibitor of migraine headache [241]. Selectively targeting the mPGES1-PGE_2_-EP1-4 arm of this pathway will likely avoid cardiovascular and GI toxicity attributed to selective targeting of COX-2 alone. By combining targeting of the mPGES1-PGE_2_-EP1-4 axis with selective adhesion-based therapy, it may be possible to significantly impact cancer prevention and therapy.

Adhesion-based therapy is generally targeted directly at surface receptors or the signal transduction pathways that mediate their activation [242]. In the case of direct integrin targeting, for example, Phase II clinical trials with cilengitide, a cyclicized arginine-glycine-aspartic acid-(RGD-)containing pentapeptide that acts as a *α*_V_ *β*3 and *α*_V_ *β*5 integrin antagonist, demonstrated clinical activity with limited side effects in glioblastoma patients [243, 244]. Based on these clinical results, the first Phase III clinical trial was initiated with an integrin antagonist [243, 244]. Similarly, *α*_5_ *β*_1_ integrins are also inhibited by the RGD amino acid sequence [245, 246], while *α*_4_ *β*_1_ are targeted by EILDV and REDV sequences [247]. In the case of cadherin targeting, ADH-1 is a cyclic pentapeptide that disrupts N-cadherin adhesion complexes that is being used to treat melanoma [248–250]. In Phase I studies ADH-1 used in combination with melphalan is well tolerated after isolated limb perfusion to treat regionally advanced melanoma. This approach using ADH-1 is believed to help overcome melanoma chemoresistance [250]. As we enter an era of personalized cancer therapy, using peptides to target specific adhesion receptors may be a viable adjuvant for selective targeting.

Targeting the signal transduction pathways downstream of adhesion receptor signaling involves a variety of molecular targets. These include the kinases, phosphatases GAP, GEF, Rho family GTPases, adapter molecules, and scaffolding proteins among others. In the case of kinase targets, Src is a good candidate [251]. Src protein family members are useful because they serve as starting points for multiple signaling cascades involved in extracellular sensory activity [251]. This class of drugs includes the following: Bosutinib, AZD0530, and Dasatinib that target both cadherin/p120 catenin which affects adherens junctions [251]. Simultaneously, these compounds can affect integrin/FAK p130Cas, paxillin, and Rho, a downstream that affects interactions with ECM [251]. Preclinically for instance, AZD053 prevents phosphorylation of paxillin and FAK and suppresses metastasis *in vivo *[251].

 Another effective kinase adhesion target is FAK [252, 253]. One of the most promising FAK inhibitors is PND-1186, which blocks FAK Tyr-397 phosphorylation [254–256]. *In vitro*, PND-1186 blocks FAK tyrosine phosphorylation while activating caspase-3 and initiating breast tumor cell apoptosis [255]. PND-1186 has also been tested *in vivo *and inhibits the growth of orthotopic breast carcinoma mouse models [254]. Targeting kinase molecules or the other intracellular signal pathway molecules may exhibit off-target effects that can be beneficial or cause unwanted side effects. Identifying patients with limited risk that will derive the most benefit from a given approach is essential to successful treatment.

In summary, targeting cell adhesion holds great promise for cancer therapy. As we learn more about individualizing cancer therapy, identifying patients that would receive the most benefit will help to direct targeting. For example, targeting specific adhesion pathways could be combined with inhibiting the mPGES1-PGE_2_-EP1-4 axis in patients that also have elevated COX-2 in their tumors or elevated PGE_2_ metabolites in their blood and/or urine. This approach may serve as an effective means of personalizing treatment or providing specifically targeted adjuvant therapy.

## Figures and Tables

**Table 1:** Prostaglandins in cancer cell adhesion, migration, and invasion summary table.

Adhesive factor	Tissue	PG	Biological effect	Refs
Cadherins				
↓E-cadherin	RIE-S	↑PGE_2_	COX-2-mediated PGE_2_ production in rat intestinal epithelial cells (RIE) downregulates E-cadherin	[88]
↓E-cadherin	SCC	↑PGE_2_	Downregulates E-cadherin through the EP2 receptor during squamous cell carcinoma (SCC) progression	[53]
↑E-cadherin	NSCLC	↓PGE_2_	S-valproate and S-diclofenac increased E-cadherin but reduced vimentin and ZEB1	[84]
↓E-cadherin	TCC	↑PGE_2_	Reciprocal correlation between cyclooxygenase-2 expression and E-cadherin in human bladder transitional cell carcinoma (TCC).	[85, 87]
↑E-cadherin	Melanoma	↑PGE_2_	Decrease of TGF*β*1-induced EMT properties in Madin-Darby canine kidney (MDCK) cells is associated with regaining E-cadherin expression	[257]
↑E-cadherin	MDCK	↑PGD_2_	Decrease of TGF*β*1-induced EMT properties in MDCK cells is associated with regaining E-cadherin expression	[258]
↓VE-cadherin	HLVE	↓PGI_2_	Inhibition of PGI_2_-mediated human lung vascular endothelial cell (HLVE) responses decreased VE-cadherin expression and increased eosinophil adhesion	[259]

Focal adhesions				
↑Actin bundles	HeLa	↑PGE_2_	Examination of cyclooxygenase-dependent actin bundles in HeLa cells.	[144]
CREB activation	Raw264.7	↑COX2	Examination of Col-I on the COX-2 expression and the signaling pathways in macrophages.	[145]
↑Focal adhesions	Osteoblasts	↑COX2	Focal adhesion promotes fluid shear stress induction of COX-2 and PGE_2_ release in osteoblasts	[146–148]
↑Focal adhesions	293-EBNA-HEK	↑PGF2*α*	Regulates Rho-mediated morphological changes	[150, 151]

Integrins				
*α*_2_ *β*_1_	Caco-2	↑LTD4/↑PGE_2_	Increased adhesion to collagen I.	[177]
*β*_1_	HT-29	↓PGE_2_	Decreased adhesion and migration on extracellular matrix	[178]
*α*_3_	Mammary TC	↓PGE_2_	Decreased adhesion to laminin	[179]
*α*_5_ *β*_1_	HLC	↑PGE_2_	Increased adhesion of human lung carcinoma (HLC) cells to fibronectin	[180]
*α*IIb*β*_3_	B16a melanoma	↑12-HETE	Increased adhesion to fibronectin, endothelial cells, and endothelial cell matrix	[182, 183]
